# Differential impact of ventricular-arterial coupling on left ventricular function in patients with acute myocardial infarction: a comparison between preserved and reduced ejection fraction

**DOI:** 10.1186/s44348-025-00045-6

**Published:** 2025-04-08

**Authors:** Hae Eun Yun, Seung-Jae Joo, Geum Ko, Ki Yung Boo, Jae-Geun Lee, Joon-Hyouk Choi, Song-Yi Kim

**Affiliations:** 1 Department of Internal Medicine, Jeju National University Hospital, Jeju, Republic of Korea; 2 Department of Internal Medicine, Jeju National University College of Medicine, Jeju, Republic of Korea

**Keywords:** Vascular stiffness, Myocardial infarction, Hemodynamics, Ventricular function

## Abstract

**Background:**

Ventricular-arterial coupling (VAC) plays a crucial role in the initiation and progression of heart failure in patients with coronary artery disease. The influence of VAC on left ventricular (LV) function may vary depending on LV systolic function. This study investigated the relationship between VAC and LV function in patients with acute myocardial infarction (AMI), stratified by ejection fraction (EF).

**Methods:**

Echocardiographic indices of LV volumes, systolic function, and diastolic function were measured using standard techniques. Effective arterial elastance (E_A_) was calculated based on stroke volume derived from the LV outflow waveform. Effective LV end-systolic elastance was determined using the single-beat method. The central aortic pressure waveform was recorded via applanation tonometry. Characteristic impedance (Zc) of the aortic root was calculated using Fourier transformation of both aortic pressure and flow waveforms.

**Results:**

A total of 85 patients (mean age, 58.5 ± 10.6 years) with AMI were enrolled. They were classified into two groups: those with reduced EF (< 50%, 27 patients) and those with preserved EF (≥ 50%, 58 PATIENTS). In the adjusted linear regression analysis, E’ velocity was significantly associated with VAC (β = –0.310, *P* = 0.008) in the preserved EF group but not in the reduced EF group. LV global longitudinal strain showed significant positive associations with VAC (β = 0.505, *P* < 0.001), E_A_ index (β = 0.536, *P* < 0.001), and Zc (β = 0.344, *P* = 0.018) exclusively in the preserved EF group.

**Conclusions:**

The distinct influence of EF status on the relationships between hemodynamic parameters and LV diastolic and systolic functions suggests a differential interplay between arterial and ventricular dynamics depending on LV systolic function.

## Background

Patients with coronary artery disease (CAD) often exhibit increased arterial stiffness due to shared common risk factors and pathophysiologic mechanisms [1, 2]. This stiffened arterial system and aorta, along with the resulting elevated systolic blood pressure (SBP), increase left ventricular (LV) afterload and myocardial oxygen demand. Simultaneously, low diastolic blood pressure (DBP) reduces coronary perfusion pressure, exacerbating myocardial ischemia. Together, these factors contribute to LV dysfunction and may ultimately lead to heart failure (HF), particularly HF with preserved ejection fraction (HFpEF) in patients with CAD [2–6].

Aortic load, which determines LV afterload, consists of both steady and dynamic components. The steady component is primarily influenced by microvascular properties, such as peripheral vascular resistance. In contrast, the dynamic component is shaped by the properties of conduit arteries, including the characteristic impedance (Zc) of the proximal aorta, the magnitude and location of wave reflections on the incident wave, and total arterial compliance—all of which are associated with arterial stiffness [4, 5, 7].

Optimal coordination between LV and the aorta is essential for maintaining efficient cardiac mechanics. Ventricular-arterial coupling (VAC) describes this interaction and has been shown to influence LV work and efficiency [4, 8]. As such, VAC serves as a valuable tool for evaluating how changes in ventricular function or arterial characteristics affect overall cardiac performance, particularly in the initiation and progression of HF [4, 5]. However, directly assessing ventricular-arterial interplay is challenging because arterial load is expressed in the frequency domain, while LV systolic function is measured in the time domain. Consequently, elastance—which describes the relationship between changes in pressure and volume and shares a common unit—has frequently been used to approximate the VAC index, despite certain limitations. VAC is typically expressed as the ratio of effective arterial elastance (E_A_) to effective left ventricular elastance (E_LV_) [4, 8].

VAC influences both LV systolic and diastolic function, with differing interactions proposed for HF with reduced ejection fraction (HFrEF) and HFpEF [4]. Consequently, the impact of VAC on LV function may differ between patients with CAD and reduced or preserved EF, yet this remains an area of limited investigation. In this study, we explored the association between VAC and LV function in patients with acute myocardial infarction (AMI), stratified by ejection fraction (EF).

## Methods

### Study patients

Patients hospitalized for AMI who underwent successful coronary reperfusion and consented to participate in this research were enrolled sequentially from June 2020 to May 2021. Patients with significant valvular heart disease or without sinus rhythm were excluded. Participants were categorized into two groups based on their EF: preserved EF (≥ 50%) and reduced EF (< 50%).

Data on age, sex, height, weight, body surface area (BSA), estimated glomerular filtration rate (eGFR), and comorbidities such as hypertension, diabetes mellitus, or angina were collected from electronic medical records. Additionally, previous medical histories of myocardial infarction (MI), HF, or stroke, smoking status, type of AMI, modality of coronary reperfusion, culprit lesions identified via coronary angiography, and discharge medications were also obtained.

### Transthoracic echocardiographic study

Transthoracic echocardiographic studies were performed using the Vivid E95 system (GE Medical) within 4 days of the index event. M-mode tracings were used to measure LV wall thickness, LV end-diastolic and end-systolic dimensions, and left atrial (LA) dimension. The LA end-systolic volume was calculated using the biplane disc method. The LV mass index was determined using the Devereux formula [9]. LV volumes during diastole and systole were assessed from apical four- and two-chamber views using the modified Simpson method, and LV EF was subsequently calculated.

Using pulsed and tissue Doppler echocardiographic imaging, standard diastolic filling parameters were measured from the apical four-chamber view. These included peak early-diastolic (E wave) and peak late-diastolic (A wave) transmitral flow velocities, the E/A ratio, early-diastolic (E’ wave), late-diastolic (A’ wave), and systolic (S’ wave) septal mitral annular velocities, as well as the E/E’ ratio. The time-velocity integral of left ventricular outflow tract (LVOT) flow, obtained via pulsed-wave Doppler echocardiography from the apical five-chamber view, was multiplied by the LVOT area, measured in the parasternal long-axis view, to calculate stroke volume (SV). Cardiac output (CO) was calculated as SV × heart rate/1,000 and indexed to BSA to derive the cardiac index (CI). The peak systolic velocity of tricuspid regurgitant flow, obtained from the continuous-wave Doppler image in the right ventricular inflow view, was used to estimate right ventricular systolic pressure (RVSP). Right atrial pressure was estimated based on the diameter of the inferior vena cava and its changes during respiration.

LV strain analysis was performed using vendor-provided software on two-dimensional speckle-tracking images from the apical two-, three-, and four-chamber views. The average LV global longitudinal strain (GLS) was then calculated.

### Hemodynamic study

Hemodynamic data were collected in the supine position immediately after the transthoracic echocardiographic study. To account for variations in body size, all hemodynamic parameters were indexed to BSA [8]. Brachial blood pressure (BP) was measured using a digital sphygmomanometer (Microlife BP A100, Microlife AG). Brachial pulse pressure (PP) was calculated as the difference between brachial SBP and DBP. Mean brachial BP was calculated using the formula: brachial PP/3 + DBP. Systemic vascular resistance (SVR) was determined by multiplying the mean brachial BP by 80, dividing by CO, and indexing the result to BSA (SVR index, SVRI). Central aortic pressures were measured using pressure wave analysis (PWA) of the radial artery pressure waveform via applanation tonometry (SphygmoCor, AtCor). The radial pressure waveform was calibrated using brachial SBP and DBP. PWA provided measurements of central (proximal aortic) SBP, central DBP, central PP, central end-systolic pressure (ESP), aortic augmentation index adjusted to a heart rate of 75 beats per minute (AIx75), pressure–time index during systole (sPTI), and pressure–time index during systole diastole (dPTI). Total arterial compliance (TAC) was calculated using the following equation and indexed to BSA (TAC index, TACI): TAC = (dPTI × SV)/[(sPTI + dPTI) × (central ESP – central DBP)] [10, 11].

### Measurements of VAC and its components

**Fig. 1:**
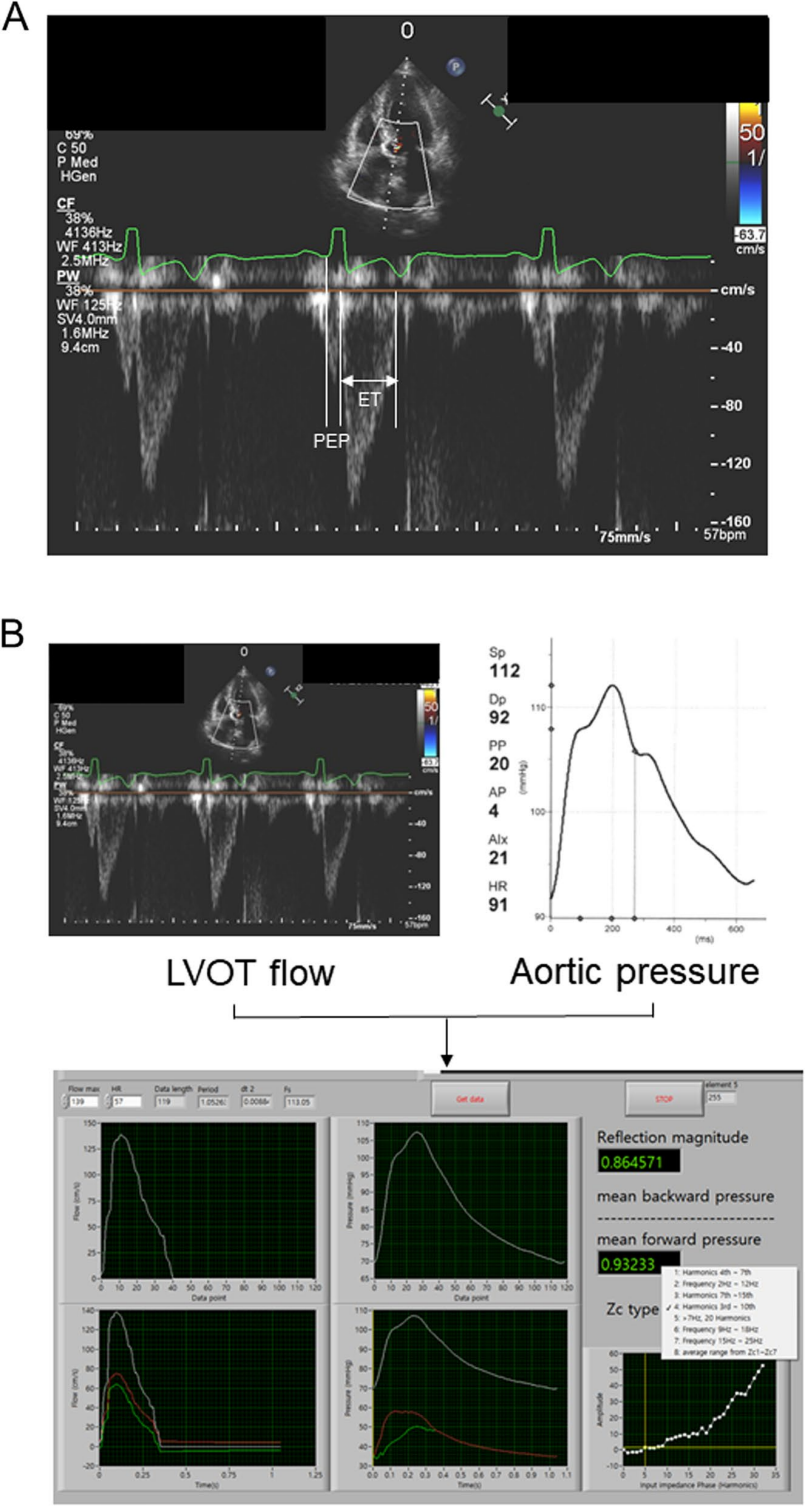
Measurements of the tNd, aortic characteristic impedance, and reflection magnitude (RM). **A** The tNd, defined as the ratio of the pre-ejection periods (PEP) to the total systolic period (PEP + ejection time [ET]) during ventricular systole, was derived from the pulsed-wave Doppler tracing of left ventricular outflow tract (LVOT) flow at the apical five-chamber view. It was calculated as the ratio of the interval from the electrocardiogram (ECG) Q wave to flow onset, to the interval from the ECG Q wave to the end of flow. **B** Measurements of aortic characteristic impedance and RM. Digitized data from LVOT flow and aortic pressure were aligned to calculate characteristic impedance and RM

Brachial ESP was calculated as 0.9 times the brachial SBP. E_A_ was determined by dividing ESP by SV and indexed to BSA as E_A_ index (E_A_I). The single-beat method was employed to estimate E_LV_ using a time-varying elastance curve and the ratio of the pre-ejection period to the total systolic period (tNd) [12–14]. The tNd was derived from pulsed-wave Doppler tracings of LVOT flow at the apical 5-chamber view as the ratio of the interval from the electrocardiogram (ECG) Q wave to flow onset to the interval from the ECG Q wave to end flow (Fig. 1A). E_LV_ was then indexed to BSA as E_LV_ index (E_LV_I). VAC was calculated as the ratio of E_A_ to E_LV_.

### Measurements of aortic characteristic impedance and reflection magnitude

Aortic pressure-flow analysis was conducted using digitized LVOT flow data obtained from pulsed-wave Doppler echocardiography at the apical five-chamber view and aortic pressure derived from the radial artery waveform. The analysis was performed using custom-developed software programmed in LabVIEW (National Instruments).

The systolic ejection time was synchronized by aligning the rapid increase in the aortic pressure wave with the onset of LVOT flow, and the dicrotic notch in the aortic pressure with the conclusion of LVOT flow. After Fourier transformation, the modulus of the aortic pressure to LVOT flow in the frequency domain was used to compute the aortic input impedance (Zin). The average value of Zin's third through tenth harmonics was then used to calculate the aortic characteristic impedance (Zc). Reflection magnitude (RM) was then computed as the ratio of the backward pressure wave to the forward pressure wave after wave separation analysis (Fig. 1B).

### Statistical analysis

Continuous variables are expressed as the median (interquartile range, IQR), while categorical variables are presented as counts (%). The Mann–Whitney U-test was employed to compare median values between patients with preserved EF and reduced EF, while the chi-square test was used to analyze categorical variables. Associations between hemodynamic data and E'velocity, E/E'ratio, and LV GLS were analyzed using correlation and linear regression methods. Statistical analyses were performed using IBM SPSS ver. 23 (IBM Corp), and a *P*-value of < 0.05 was considered statistically significant.

## Results

A total of 90 patients with AMI were enrolled sequentially. After excluding 5 patients with severe aortic stenosis, 85 participants remained in the study. Of these, 27 patients with reduced EF were categorized into the reduced EF group, while the remaining 58 were assigned to the preserved EF group.

### Baseline clinical characteristics

Age, sex, height, body mass index, BSA, comorbidities including hypertension, diabetes mellitus, and angina, prior medical history of MI, HF, or stroke, current smoking status, initial Killip class, and eGFR showed no significant differences between patients with reduced EF and those with preserved EF. The percentage of patients with ST-elevation MI was higher in the reduced EF group compared to the preserved EF group. Except for one patient in the preserved EF group who underwent only percutaneous balloon angioplasty, all patients received percutaneous coronary stent placement for coronary reperfusion. Compared to the preserved EF group, the reduced EF group had a higher proportion of culprit lesions in the left anterior descending artery. Medications at discharge, including antiplatelet agents, beta-blockers, renin-angiotensin system inhibitors, calcium channel blockers, nitrates, and statins, were administered similarly across both groups (Table 1).

**Table 1:** Baseline characteristics of patients

Characteristic	Total (n = 85)	Reduced EF (n = 27)	Preserved EF (n = 58)	*P*-value
Age (yr)	60 (51–66)	58 (50–66)	61 (52–65)	0.467
Male sex	76 (89.4)	25 (92.6)	51 (87.9)	0.712
Height (cm)	168 (163–173)	168 (163–174)	168 (163–173)	0.698
Body mass index (kg/m^2^)	25.2 (23.5–28.1)	26.0 (23.5–28.1)	25.1 (23.5–28.2)	0.527
Body surface area (/m^2^)	1.83 (1.72–1.93)	1.83 (1.78–1.97)	1.81 (1.70–1.93)	0.422
Hypertension	32 (37.6)	10 (37.0)	22 (37.9)	> 0.999
Diabetes mellitus	29 (34.1)	10 (37.0)	19 (32.8)	0.807
Angina	10 (11.8)	2 (7.4)	8 (13.8)	0.492
Prior MI	5 (5.9)	1 (3.7)	4 (6.9)	> 0.999
Prior heart failure	1 (1.2)	0 (0)	1 (1.7)	> 0.999
Stroke	5 (5.9)	1 (3.7)	4 (6.9)	> 0.999
Smoker	45 (52.9)	18 (66.7)	27 (46.6)	0.105
Killip class ≥ 2	8 (9.4)	5 (18.5)	3 (5.2)	0.133
eGFR	86.1 (76.7–103.1)	81.2 (75.6–91.8)	93.5 (78.7–106.9)	0.080
eGFR < 60 mL/min/1.73 m^2^	8 (9.4)	1 (3.7)	7 (12.1)	0.426
ST-elevation MI	47 (55.3)	20 (74.1)	27 (46.6)	0.021
PCI	85 (100)	27 (100)	58 (100)	
PCI with stents	84 (98.8)	27 (100)	57 (98.3)	> 0.999
Culprit lesion				0.008
Left main	2 (2.3)	1 (3.7)	1 (1.7)	
Left anterior descending artery	43 (50.6)	20 (74.1)	23 (39.7)	
Left circumflex artery	10 (11.8)	0 (0)	10 (17.2)	
Right coronary artery	30 (35.3)	6 (22.2)	24 (41.4)	
Medication				
Aspirin	83 (97.6)	26 (96.3)	57 (98.3)	0.537
P2Y12 inhibitor	85 (100)	27 (100)	58 (100)	-
β-Blocker	71 (83.5)	25 (92.6)	46 (79.3)	0.208
ACEI	3 (3.5)	1 (3.7)	2 (3.4)	> 0.999
ARB	39 (45.9)	15 (55.6)	24 (41.4)	0.250
Calcium channel blocker	10 (11.8)	3 (11.1)	7 (12.1)	> 0.999
Nitrate	8 (9.4)	1 (3.7)	7 (12.1)	0.426
Statin	85 (100)	27 (100)	58 (100)	-

Values are presented as median (interquartile range) or number (%)

*EF* ejection fraction, *MI* myocardial infarction, *eGFR* estimated glomerular filtration rate, *PCI* percutaneous coronary intervention, *ACEI* angiotensin-converting enzyme inhibitor, *ARB* angiotensin receptor blocker

### Comparison of echocardiographic and hemodynamic data

LV wall thickness, LV end-diastolic dimensions, LV mass index, LA dimension, and LA end-systolic volume index did not differ significantly between the preserved EF and reduced EF groups. However, the reduced EF group showed greater LV end-systolic dimensions, end-diastolic volume, end-systolic volume, and LV GLS, along with lower relative wall thickness, SV, CO, CI, and EF. E’ velocity, S’ velocity, and A velocity were significantly lower in the reduced EF group, while other LV diastolic parameters, including E/E’ ratio and RVSP, were comparable between the two groups (Table 2).

**Table 2:** Comparison of echocardiographic data

Variable	Total (n = 85)	Reduced EF (n = 27)	Preserved EF (n = 58)	*P*-value
IVS thickness (cm)	1.01 (0.95 to 1.08)	1.02 (0.95 to 1.13)	1.01 (0.94 to 1.06)	0.737
LVPW thickness (cm)	0.93 (0.84 to 0.98)	0.89 (0.80 to 0.97)	0.95 (0.88 to 0.99)	0.078
LV end-diastolic dimension (cm)	4.84 (4.57 to 5.10)	4.89 (4.63 to 5.13)	4.80 (4.56 to 5.08)	0.345
LV end-systolic dimension (cm)	3.30 (2.94 to 3.61)	3.65 (3.19 to 3.91)	3.24 (2.78 to 3.39)	< 0.001
LV mass index (g/m^2^)	90.6 (81.4 to 101.3)	89.6 (81.0 to 102.9)	90.8 (81.1 to 101.0)	0.858
Relative wall thickness	0.38 (0.36 to 0.40)	0.37 (0.33 to 0.40)	0.39 (0.37 to 0.41)	0.016
LA dimension (cm)	4.14 (3.94 to 4.34)	4.06 (3.86 to 4.27)	4.18 (4.01 to 4.43)	0.073
LA end-systolic volume index (mL/m^2^)	35.4 (31.3 to 41.9)	36.6 (30.3 to 42.5)	35.3 (31.8 to 41.8)	0.891
End-diastolic volume (mL)	110.9 (85.3 to 132.3)	126.9 (101.2 to 144.2)	104.3 (83.6 to 126.4)	0.017
End-systolic volume (mL)	49.0 (40.4 to 61.7)	70.1 (55.9 to 80.8)	43.2 (37.2 to 54.8)	< 0.001
EF (%)	53.0 (48.2 to 59.8)	46.0 (43.1 to 48.1)	58.9 (52.9 to 61.2)	< 0.001
LV GLS (%)	–14.4 (–15.8 to –11.4)	–10.7 (–12.2 to –7.9)	–15.1 (–16.5 to –13.8)	< 0.001
LVOT diameter (cm)	2.13 (2.04 to 2.23)	2.17 (2.09 to 2.24)	2.12 (2.02 to 2.22)	0.301
Stroke volume (mL)	58.5 (49.3 to 67.2)	50.9 (44.7 to 58.0)	61.9 (54.9 to 74.0)	< 0.001
Heart rate (beats/min)	73 (63 to 82)	76 (66 to 81)	71 (62 to 82)	0.286
Cardiac output (L/min)	4.25 (3.67 to 4.88)	3.86 (3.36 to 4.33)	4.45 (3.80 to 5.00)	0.003
Cardiac index (L/min/m^2^)	2.28 (2.02 to 2.75)	2.08 (1.83 to 2.51)	2.44 (2.10 to 2.85)	0.002
E velocity (cm/sec)	54.2 (44.2 to 66.0)	52.3 (37.5 to 66.5)	55.3 (45.1 to 65.5)	0.227
A velocity (cm/sec)	70.1 (57.8 to 81.2)	63.2 (53.1 to 73.1)	74.0 (63.2 to 87.9)	0.007
E/A ratio	0.71 (0.61 to 1.06)	0.72 (0.56 to 1.09)	0.69 (0.61 to 1.06)	0.832
E’ velocity (cm/sec)	5.74 (5.01 to 6.42)	5.31 (4.78 to 5.74)	6.08 (5.08 to 7.30)	0.004
A’ velocity (cm/sec)	9.55 (8.24 to 11.00)	9.67 (7.47 to 10.52)	9.54 (8.35 to 11.10)	0.558
S’ velocity (cm/sec)	7.37 (6.14 to 8.49)	6.51 (6.00 to 7.72)	7.65 (6.38 to 8.79)	0.042
E/E’ ratio	9.0 (7.3 to 11.7)	9.8 (7.2 to 12.4)	8.6 (7.4 to 10.9)	0.372
RVSP (mmHg)	24.1 (22.3 to 28.6)	23.9 (22.5 to 27.9)	25.7 (22.2 to 29.5)	0.273

Values are presented as median (interquartile range)

*EF* ejection fraction, *IVS* interventricular septum, *LVPW* left ventricular posterior wall, *LV* left ventricular, *LA* left atrial, *GLS* global longitudinal strain, *LVOT* left ventricular outflow tract, *RVSP* right ventricular systolic pressure

Brachial and central PP was significantly lower in the reduced EF group. However, brachial and central BP, SVRI, TACI, and AIx75 did not differ significantly between the groups. Similarly, E_LV_I showed no significant differences. E_A_I were significantly higher in the reduced EF group (median, 1.15 mmHg/mL∙m^2^ [IQR, 0.90–1.37 mmHg/mL∙m^2^] vs. median, 1.00 mmHg/mL∙m^2^ [IQR, 0.76–1.16 mmHg/mL∙m^2^], *P* = 0.039), contributing to an increased VAC (median, 1.13 [IQR, 1.01–1.30] vs. median, 0.97 [IQR, 0.85–1.20], *P* = 0.003). There were no significant differences in Zc or RM between the groups (Table 3).

**Table 3:** Comparison of hemodynamic data

Variable	Total (n = 85)	Reduced EF (n = 27)	Preserved EF (n = 58)	*P*-value
Brachial SBP (mmHg)	120 (110–129)	116 (104–127)	124 (113–130)	0.069
Brachial DBP (mmHg)	74 (68–83)	74 (68–83)	75 (67–83)	0.843
Brachial PP (mmHg)	45 (38–54)	38 (33–57)	47 (40–54)	0.029
Central SBP (mmHg)	110 (100–119)	106 (95–114)	113 (101–120)	0.058
Central DBP (mmHg)	75 (68–84)	75 (68–84)	77 (68–83)	0.895
Central PP (mmHg)	33 (27–44)	28 (22–45)	35 (29–44)	0.018
Heart rate (beats/min)	70 (62–80)	75 (64–82)	67 (61–78)	0.092
SVRI (dynes/sec/cm^−7^)	942 (798–1,096)	1,049 (801–1,228)	928 (788–1,020)	0.091
TACI (mL/mmHg∙m^2^)	0.88 (0.61–1.09)	0.83 (0.61–1.09)	0.90 (0.62–1.10)	0.467
AIx75 (%)	21.2 (14.0–25.9)	21.2 (15.0–26.3)	21.2 (13.5–25.8)	0.741
E_LV_I (mmHg/mL∙m^2^)	0.95 (0.82–1.18)	1.00 (0.79–1.22)	0.95 (0.83–1.17)	0.985
E_A_I (mmHg/mL∙m^2^)	1.05 (0.79–1.23)	1.15 (0.90–1.37)	1.00 (0.76–1.16)	0.039
Ventricular-arterial coupling	1.03 (0.89–1.23)	1.13 (1.01–1.30)	0.97 (0.85–1.20)	0.003
Zc (dyne-sec/cm^3^)	197 (136–278)	190 (120–275)	208 (139–283)	0.406
Reflection magnitude	0.84 (0.80–0.89)	0.86 (0.81–0.93)	0.84 (0.78–0.88)	0.116

Values are presented as median (interquartile range)

*EF* ejection fraction, *SBP* systolic blood pressure, *DBP* diastolic blood pressure, *PP* pulse pressure, *SVRI* systemic vascular resistance index, *TACI* total arterial compliance index, *AIx75* augmentation index adjusted to a heart rate of 75 beats per minute, *E*_LV_*I* left ventricular end-systolic elastance index, *E*_A_*I* effective arterial elastance index, *Zc* characteristic impedance

### Associations of E’ velocity and LV GLS with hemodynamic data

**Fig. 2:**
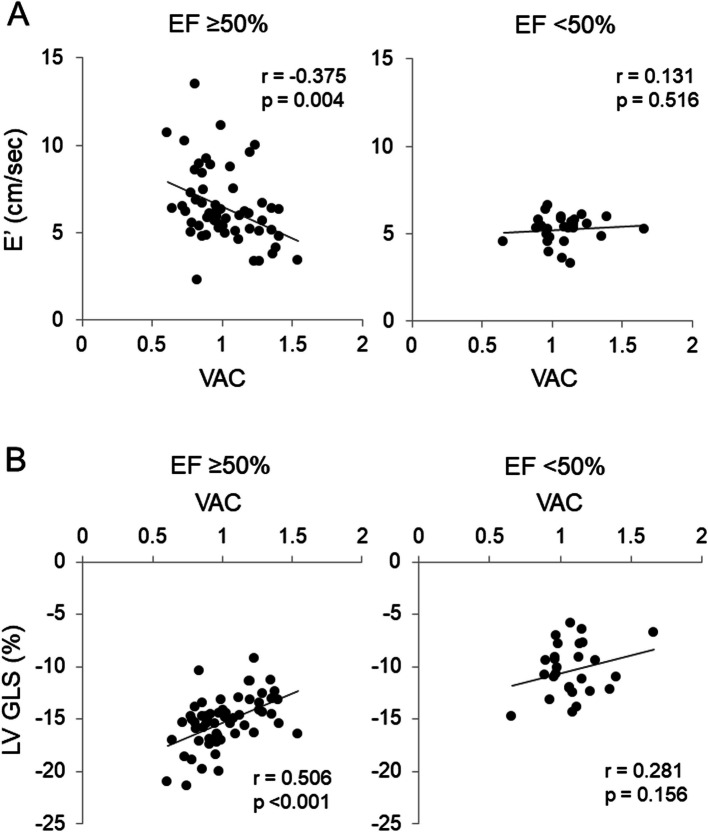
Correlations of ventricular-arterial coupling (VAC) with E’ velocity and left ventricular (LV) global longitudinal strain (GLS) stratified by ejection fraction (EF). The relationship between VAC and E’ velocity in patients with (**A**) EF ≥ 50% and (**B**) EF < 50%. The relationship between VAC and LV GLS in patients with (**C**) EF ≥ 50% and (**D**) EF < 50%

A distinct pattern of association between E’ velocity and hemodynamic parameters was observed based on EF status. In the preserved EF group, E’ velocity demonstrated a significant negative correlation with E_A_I, VAC, and Zc, and after adjusting for age, sex, and height in linear regression analysis, E’ velocity remained significantly associated only with VAC (β = –0.310, *P* = 0.008) (Fig. 2A), whereas no significant associations were observed with E_A_I or Zc (Tables 4, 5). In contrast, no significant correlation was found between E’ velocity and VAC in the reduced EF group (Fig. 2B). Although a correlation between E’ velocity and E_LV_I was identified in the reduced EF group, this association did not remain significant after adjustment in the linear regression analysis. Furthermore, no significant associations were observed between E’ velocity and SVRI, TACI, E_LV_I, or RM in either group after adjusted linear regression analysis (Table 5).

**Table 4:** Correlations of E’ velocity, E/E’ ratio and LV GLS with hemodynamic data

Variable	E’ velocity			E/E’ ratio			LV GLS		
	All	Reduced EF	Preserved EF	All	Reduced EF	Preserved EF	All	Reduced EF	Preserved EF
SVRI	–0.263^*^	–0.275	–0.246	0.050	0.103	–0.006	0.290^*^	0.083	0.282^*^
TACI	0.143	–0.029	0.146	–0.198	–0.224	–0.182	–0.163	0.239	–0.278^*^
E_LV_I	–0.036	–0.409^*^	0.017	0.026	–0.099	0.075	0.000	0.002	0.009
E_A_I	–0.300^*^	–0.284	–0.272^*^	0.101	0.134	0.071	0.381^*^	0.210	0.394^*^
VAC	–0.351^*^	0.131	–0.375^*^	0.106	0.405^*^	–0.044	0.521^*^	0.281	0.506^*^
Zc	–0.179	0.054	–0.278^*^	0.225^*^	0.220	0.235	0.039	–0.355	0.341^*^
RM	–0.135	0.009	–0.131	–0.067	–0.200	–0.016	0.189	0.468^*^	–0.070

*LV* left ventricular, *GLS* global longitudinal strain, *SVRI* systemic vascular resistance index, *TACI* total arterial compliance index, *E*_LV_*I* left ventricular end-systolic elastance index, *E*_A_*I* effective arterial elastance index, *VAC* ventricular-arterial coupling, *Zc* characteristic impedance; RM, reflection magnitude

^*^P < 0.05

**Table 5:** Linear regression analysis of E’ velocity and LV GLS with hemodynamic data

Variable	Preserved EF				Reduced EF			
	Unadjusted		Adjusted^a^		Unadjusted		Adjusted^a^	
	β	*P*-value	β	*P*-value	β	*P*-value	β	*P*-value
E’ velocity								
SVRI	–0.246	0.062	–0.072	0.576	–0.275	0.165	–0.127	0.552
TACI	0.146	0.274	0.027	0.824	–0.029	0.885	–0.118	0.553
E_LV_I	0.017	0.900	0.187	0.183	–0.409	0.034	–0.243	0.318
E_A_I	–0.272	0.039	–0.128	0.347	–0.284	0.151	–0.071	0.762
VAC	–0.375	0.004	–0.310	0.008	0.131	0.516	0.168	0.392
Zc	–0.278	0.034	–0.151	0.243	0.054	0.788	0.050	0.799
RM	–0.131	0.326	–0.187	0.122	0.009	0.965	0.100	0.626
LV GLS								
SVRI	0.282	0.032	0.325	0.026	0.083	0.680	0.008	0.732
TACI	–0.278	0.034	–0.290	0.037	0.239	0.231	0.235	0.273
E_LV_I	0.009	0.949	0.067	0.679	0.002	0.993	0.016	0.952
E_A_I	0.394	0.002	0.536	< 0.001	0.210	0.293	0.298	0.234
VAC	0.506	< 0.001	0.505	< 0.001	0.281	0.156	0.271	0.199
Zc	0.341	0.009	0.344	0.018	–0.355	0.069	–0.338	0.105
RM	–0.070	0.603	–0.046	0.745	0.468	0.014	0.545	0.009

*LV* left ventricular, *GLS* global longitudinal strain, *EF* ejection fraction, *SVRI* systemic vascular resistance index, *TACI* total arterial compliance index, *E*_LV_*I* left ventricular end-systolic elastance index, *E*_A_*I* effective arterial elastance index, *VAC* ventricular-arterial coupling, *Zc* characteristic impedance, *RM* reflection magnitude

^a^Adjusted for age, sex, and height

The E/E’ ratio, although correlated with VAC in the reduced EF group, did not show any significant associations with hemodynamic parameters in both groups after adjusted linear regression analysis.

**Fig. 3:**
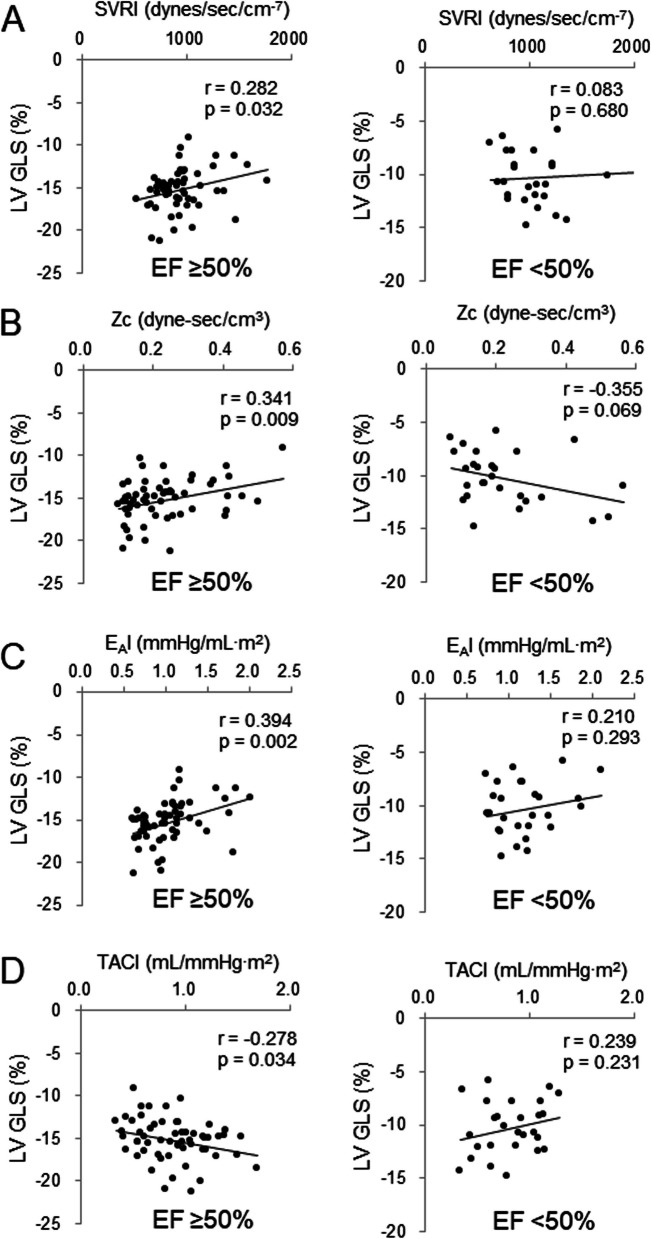
Correlations of hemodynamic parameters with left ventricular (LV) global longitudinal strain (GLS) stratified by ejection fraction (EF). The relationship between systemic vascular resistance index (SVRI) and LV GLS in patients with (**A**) EF ≥ 50% and (**B**) EF < 50%. The relationship between characteristic impedance (Zc) and LV GLS in patients with (**C**) EF ≥ 50% and (**D**) EF < 50%. The relationship between effective arterial elastance index (E_A_I) and LV GLS in patients with (**E**) EF ≥ 50% and (**F**) EF < 50%. The relationship between total arterial compliance index (TACI) and LV GLS in patients with (**G**) EF ≥ 50% and (H) EF < 50%

Similarly, a distinct pattern of association between LV GLS and hemodynamic parameters emerged based on EF status. In the preserved EF group, LV GLS exhibited a significant positive correlation with VAC (Fig. 2C), SVRI (Fig. 3A), Zc (Fig. 3C), and E_A_I (Fig. 3E) as well as a significant negative correlation with TACI (Fig. 3G). After adjusting for age, sex, and height in linear regression analysis, LV GLS remained significantly associated with VAC (β = 0.505, *P* < 0.001), along with SVRI (β = 0.325, *P* = 0.026), E_A_I (β = 0.536, *P* < 0.001), Zc (β = 0.344, *P* = 0.018), and TACI (β = –0.290, *P* = 0.037) (Tables 4, 5). However, in the reduced EF group, no significant associations between LV GLS and hemodynamic parameters were observed in this group (Fig. 2D, 3B, 3D, 3 F, 3H), except for a positive correlation with RM (β = 0.545, *P* = 0.009).

## Discussions

The main findings of this study indicate that VAC was significantly associated with E’ velocity and LV GLS in AMI patients with preserved EF, whereas no such association was observed in those with reduced EF. Furthermore, steady and dynamic aortic loads were found to correlate with LV GLS exclusively in AMI patients with preserved EF. These results underscore the distinct influence of EF status on the relationships between hemodynamic parameters and LV diastolic and systolic functions, as represented by E’ velocity and LV GLS, suggesting a differential interplay between arterial and ventricular dynamics in patients with preserved versus reduced EF.

In patients with HFrEF, reduction in LV pump efficiency, LV elastance, and peripheral tissue perfusion trigger compensatory activation of the sympathetic nervous system and the renin-angiotensin system, leading to elevated intravascular volume and arterial pressure, which subsequently increase arterial elastance [4]. Consequently, VAC also rises. In contrast, while ventricular-arterial mismatch is recognized as a critical factor in the onset and progression of HFpEF [5], both LV stiffness and aortic stiffness increase without a corresponding change in VAC [4]. Aligning with these observations, this study demonstrated higher E_A_I and VAC in AMI patients with reduced EF compared to those with preserved EF.

The most commonly used noninvasive method to evaluate diastolic dysfunction is the measurement of E’ velocity at the mitral annulus [15, 16]. A reduced E’ velocity serves as a well-established marker for identifying LV diastolic dysfunction, even in its earliest stages. A previous study demonstrated a significant negative association between VAC and E’ velocity in women with hypertension [7]. In this study, we found that in AMI patients with preserved EF, VAC was negatively associated with E’ velocity, while aortic steady and dynamic components showed no significant correlation. These findings suggest that ventricular-arterial mismatch exerts a more critical influence in LV diastolic dysfunction than aortic load alone. Conversely, in AMI patients with reduced EF, neither aortic load nor VAC was associated with E’ velocity, indicating that VAC exerts minimal influence on LV diastolic function in the setting of impaired LV systolic function.

LV GLS, derived from speckle-tracking echocardiography, has been shown to detect subtle changes in myocardial longitudinal fibers caused by ischemia in patients with preserved EF [17, 18]. As a result, LV GLS serves as a more sensitive marker of LV systolic dysfunction [19] and has demonstrated prognostic significance, with LV GLS > –14% identified as a poor prognostic factor in AMI patients with preserved EF [20]. Furthermore, because EF is one of the parameters used to calculate E_LV_ in this study, LV GLS was employed to investigate the relationship of LV systolic dysfunction with aortic elastance, LV elastance, and VAC. A significant positive correlation of VAC with LV GLS has been shown in women with hypertension in a previous study [7]. In this study, a significant positive association of LV GLS with SVRI, Zc, E_A_I, and VAC, as well as a significant negative association with TACI, was demonstrated in the preserved EF group, but not in the reduced EF group. These findings suggest that aortic load and ventricular-arterial mismatch may contribute to LV systolic dysfunction and the progression to HFrEF in patients with preserved EF. However, in patients with already reduced EF, the influence of aortic load and ventricular-arterial mismatch on LV systolic function appears to be minimal. These findings underscore the importance of measuring aortic load and VAC in AMI patients with preserved EF to help prevent progression to HFrEF. Since antihypertensive therapy in patients with hypertension has been shown to reduce arterial stiffness and improve VAC [21], our findings further suggest the need for stricter BP control in AMI patients with hypertension.

Augmentation index (AIx) has frequently been used to assess the contribution of the reflected wave on central systolic pressure and PP in a stiff arterial system [22]. However, AIx has been shown to be influenced by factors such as heart rate, an individual’s height, and the location of the reflected wave, rather than just its amplitude [3]. As a result, its usefulness as a predictive factor for clinical events is limited. Another index of arterial stiffness, RM, has been proposed as a more accurate and novel measure for assessing pulsatile aortic afterload on LV function, and its predictive role in HF within the general population has also been demonstrated [23, 24], although it requires complex separation of incident and reflected waves from both flow and pressure waveforms. In this study, a significant positive correlation between RM and LV GLS was observed only in the reduced EF group, suggesting a potential role for RM as an exacerbating factor in LV systolic function in AMI patients with reduced EF.

This study has several limitations. First, as a cross-sectional study analyzing the relationship between hemodynamic parameters and LV functional parameters, this research cannot establish a direct causal effect of VAC and aortic load on LV diastolic and systolic functions. Furthermore, the study only included patients in the acute stage of MI, and the impact of VAC and aortic load on LV function may differ in the chronic stage. Therefore, the findings of this study should be interpreted with caution. These limitations underscore the need for a prospective study to confirm causality and to evaluate the effect of VAC and aortic load on LV function during the chronic stage of MI. Second, while noninvasive, transfer-function-based estimation of central aortic pressure from radial artery applanation tonometry is commonly used in clinical practice, it may not always accurately capture the central aortic pressure waveform due to inherent limitations of the technique [25]. Third, patients were on medications that could influence hemodynamic parameters. However, these medications were equally distributed between the preserved and reduced EF groups. Fourth, because body size can significantly affect hemodynamic parameters, these were indexed by BSA, and height was used as an adjusting factor in the regression analysis. However, this adjustment may not fully account for the effect of body size on the association between hemodynamic parameters and LV functional parameters. Fifth, LV diastolic function was evaluated using only septal E’ velocity, which may be influenced by regional wall motion abnormalities in the basal septal myocardium. This limitation particularly could affect the relationship between E’ velocity and hemodynamic parameters especially in AMI patients with reduced EF. Sixth, this study included only AMI patients. Therefore, the influence of EF status on the relationships between hemodynamic parameters and LV diastolic and systolic function needs to be evaluated in patients with HFpEF and HFrEF due to causes other than AMI.

## Conclusions

VAC was significantly associated with E’ velocity and LV GLS in AMI patients with preserved EF, whereas no such associations were observed in those with reduced EF. Furthermore, steady and dynamic aortic loads correlated with LV GLS exclusively in AMI patients with preserved EF. These findings highlight the distinct influence of EF status on the relationships between hemodynamic parameters and LV diastolic and systolic functions, suggesting a differential interplay between arterial and ventricular dynamics depending on LV systolic function.

## Data Availability

The data underlying this article will be shared on reasonable request to the corresponding author.

## Abbreviations

Aix: Augmentation index
AIx75: Augmentation index adjusted to a heart rate of 75 beats per minute
AMI: Acute myocardial infarction
BP: Blood pressure
BSA: Body surface area
CAD: Coronary artery disease
CI: Cardiac index
COL: Cardiac output
DBP: Diastolic blood pressure
dPTI: Pressure–time indexes during diastole
E_A_: Effective arterial elastance
E_A_I: Effective arterial elastance index
ECG: Electrocardiogram
EF: Ejection fraction
eGFR: Estimated glomerular filtration rate
E_LV_: Effective left ventricular elastance
E_LV_I: Effective left ventricular elastance index
ESP: End-systolic pressure
GLS: Global longitudinal strain
HF: Heart failure
HFpEF: Heart failure with preserved ejection fraction
HFrEF: Heart failure with reduced ejection fraction
IQR: Interquartile range
LA: Left atrial
LV: Left ventricular
LVOT: Left ventricular outflow tract
MI: Myocardial infarction
PP: Pulse pressure
PWA: Pressure wave analysis
RM: Reflection magnitude
RVSP: Right ventricular systolic pressure
SBP: Systolic blood pressure
sPTI: Pressure–time indexes during systole
SV: Stroke volume
SVR: Systemic vascular resistance
SVRI: Systemic vascular resistance index
TAC: Total arterial compliance
TACI: Total arterial compliance index
tNd: Ratio of the pre-ejection period to the total systolic period
VAC: Ventricular-arterial coupling
Zc: Characteristic impedance
Zin: Input impedance
